# Systemic IL-27 administration prevents abscess formation and osteolysis via local neutrophil recruitment and activation

**DOI:** 10.1038/s41413-022-00228-7

**Published:** 2022-08-26

**Authors:** Yugo Morita, Motoo Saito, Javier Rangel-Moreno, Anthony M. Franchini, John R. Owen, John C. Martinez, John L. Daiss, Karen L. de Mesy Bentley, Stephen L. Kates, Edward M. Schwarz, Gowrishankar Muthukrishnan

**Affiliations:** 1 Center for Musculoskeletal Research, University of Rochester Medical Center, Rochester, NY USA; 2 Division of Allergy, Immunology and Rheumatology, Department of Medicine, University of Rochester Medical Center, Rochester, NY USA; 3 Department of Environmental Medicine, University of Rochester School of Medicine and Dentistry, Rochester, NY USA; 4 Department of Orthopedic Surgery, Virginia Commonwealth University, Richmond, VA USA; 5 Department of Orthopedics, University of Rochester Medical Center, Rochester, NY USA; 6 Department of Pathology and Laboratory Medicine, University of Rochester Medical Center, Rochester, NY USA

**Keywords:** Pathogenesis, Bone

## Abstract

Interleukin-27 is a pleiotropic cytokine whose functions during bacterial infections remain controversial, and its role in patients with *S. aureus* osteomyelitis is unknown. To address this knowledge gap, we completed a clinical study and observed elevated serum IL-27 levels (20-fold higher, *P* < 0.05) in patients compared with healthy controls. Remarkably, IL-27 serum levels were 60-fold higher in patients immediately following septic death than in uninfected patients (*P* < 0.05), suggesting a pathogenic role of IL-27. To test this hypothesis, we evaluated *S. aureus* osteomyelitis in WT and IL-27Rα^−/−^ mice with and without exogenous IL-27 induction by intramuscular injection of rAAV-IL-27p28 or rAAV-GFP, respectively. We found that IL-27 was induced at the surgical site within 1 day of *S. aureus* infection of bone and was expressed by M0, M1 and M2 macrophages and osteoblasts but not by osteoclasts. Unexpectedly, exogenous IL-27p28 (~2 ng·mL^−1^ in serum) delivery ameliorated soft tissue abscesses and peri-implant bone loss during infection, accompanied by enhanced local IL-27 expression, significant accumulation of RORγt^+^ neutrophils at the infection site, a decrease in RANK^+^ cells, and compromised osteoclast formation. These effects were not observed in IL-27Rα^−/−^ mice compared with WT mice, suggesting that IL-27 is dispensable for immunity but mediates redundant immune and bone cell functions during infection. In vitro studies and bulk RNA-seq of infected tibiae showed that IL-27 increased *nos1, nos2*, *il17a*, *il17f*, and *rorc* expression but did not directly stimulate chemotaxis. Collectively, these results identify a novel phenomenon of IL-27 expression by osteoblasts immediately following *S. aureus* infection of bone and suggest a protective role of systemic IL-27 in osteomyelitis.

## Introduction

Despite significant medical advances, deep bone infections continue to be the bane of orthopedic surgery, with infection rates essentially remaining at 1%–2% after elective surgery over the past 50 years^1–4^. *Staphylococcus aureus* is the major pathogen in orthopedic infections. It is responsible for 10 000–20 000 prosthetic joint infections (PJIs) annually in the United States^5,6^ and 30%–42% of fracture-related infections (FRIs)^7,8^. Unfortunately, these difficult-to-treat *S. aureus* bone infections are associated with poor clinical outcomes and high recurrence rates following revision surgery^9,10^. With the increasing incidence of methicillin-resistant *S. aureus* (MRSA) osteomyelitis and emerging strains with pan-drug resistance^11,12^, there is an urgent need for novel immunotherapies to supplement existing antibiotic therapies.

*S. aureus* causes the most lethal form of human sepsis, with a 10% mortality rate, and a catastrophic outcome of osteomyelitis is death due to sepsis and multiple organ failure^13,14^. The cellular and molecular mechanisms underlying *S. aureus* osteomyelitis-induced sepsis are largely unknown. Interestingly, several studies have reported elevated serum IL-27 levels during sepsis, suggesting that IL-27 could potentially be useful in predicting sepsis-driven mortality^15–20^. IL-27 is a heterodimeric cytokine belonging to the IL-12 cytokine family and is mainly produced by antigen-presenting cells such as macrophages, monocytes, and dendritic cells^21,22^. IL-27 is composed of the IL-27p28 and EBI3 subunits and signals through a heterodimeric cell surface receptor composed of IL-27 receptor α (IL-27Rα) and gp130^21–23^. Similar to IL-12, IL-27 signaling is mediated mainly through the intracellular JAK-STAT pathway and participates in multiple immunoregulatory activities^21–23^. Classical IL-27 signaling downregulates Th17 differentiation, stimulates regulatory T-cell development, and drives IL-10 production by CD^+^ T cells^21,22,24,25^. Studies involving cecal ligation and puncture (CLP)-induced bacterial sepsis and *S. aureus* pneumonia following influenza demonstrated that IL-27 mediates enhanced susceptibility to infection by attenuating Th17 immunity and promoting IL-10 induction^26,27^. These studies highlight the importance of IL-27 in immune suppression. On the other hand, IL-27 has been reported to promote the proliferation and differentiation of hematopoietic stem cells^28^, increase the production of proinflammatory cytokines by monocytes^29,30^, and induce Th1 differentiation^31^. Currently, the contribution of IL-27 to host immunity during *S. aureus* osteomyelitis is unknown. Here, we examined whether IL-27 is an essential cytokine involved in the pathogenesis of *S. aureus* osteomyelitis. We report that IL-27 expression was induced in patients with *S. aureus* osteomyelitis and that elevated serum IL-27 correlated with septic death in these patients. IL-27 is produced early in murine *S. aureus* bone infection, especially by macrophages and, surprisingly, by osteoblasts. We also unexpectedly discovered that exogenous and prophylactic administration of IL-27 significantly accelerates *S. aureus* clearance in mouse bone by enhancing early local host innate immune responses and preventing bone erosion.

## Results

### Serum IL-27 levels were associated with *S. aureus* osteomyelitis in patients

**Fig. 1:**
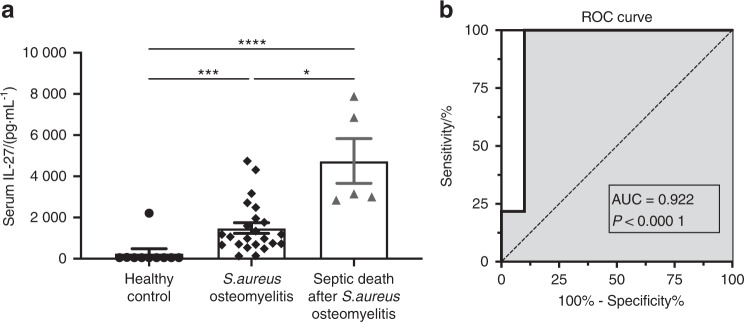
Serum IL-27 levels are elevated in patients with *S. aureus* infection. **a** Serum samples were collected from healthy individuals (*n* = 10), orthopedic patients with culture-confirmed *S. aureus* bone infections (*n* = 23), and patients who died from septic *S. aureus* osteomyelitis (*n* = 5). Serum IL-27 levels were determined via a Luminex assay. The data for each sample are presented as the mean ± SEM for each experimental group. **b** The Luminex data were utilized to generate a receiver operating characteristic (ROC) curve, and the areas under the curve (AUC) for the controls and infected patients are presented. Note that the serum IL-27 level is highly predictive of *S. aureus* osteomyelitis. The dashed line represents a nondiscriminatory test with equal sensitivity and specificity (*P < 0.05, ***P < 0.001, ****P < 0.000 1)

To better understand the involvement of IL-27 in host immune responses to *S. aureus* osteomyelitis, we measured IL-27 in serum from healthy individuals, orthopedic patients with culture-confirmed *S. aureus* bone infections, and patients who died from sepsis associated with *S. aureus* osteomyelitis. Serum IL-27 levels were significantly elevated in infected patients compared to uninfected individuals (20-fold higher, *P* < 0.05). Remarkably, IL-27 levels immediately following septic death were 60-fold higher than those in healthy individuals (Fig. 1a, *P* < 0.05), suggesting that IL-27 could be useful in predicting *S. aureus* osteomyelitis-induced septic death. Indeed, in this small patient cohort, formal analyses of IL-27 as a diagnostic biomarker using receiver operator characteristic (ROC) curve analysis revealed good prediction accuracy for identifying *S. aureus* osteomyelitis, as indicated by the area under the curve (AUC) of 0.922 (Fig. 1b, *P* < 0.000 1).

### IL-27R is required for *S. aureus*-driven early IL-27 expression in mice

**Fig. 2:**
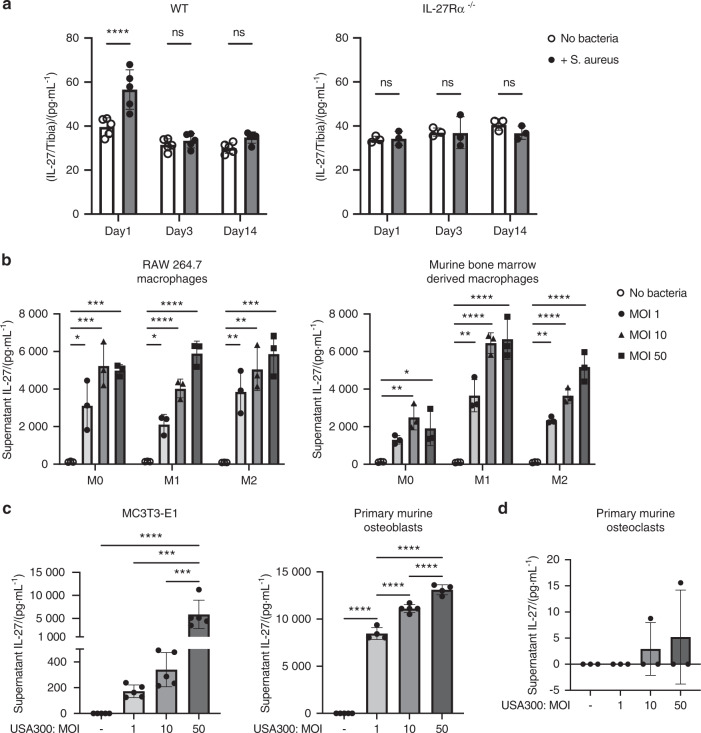
Early autoregulation of IL-27 at the infection site and evidence of MRSA-induced secretion of IL-27 by macrophages and osteoblasts but not by osteoclasts. **a** Female WT and IL-27Rα^−/−^ mice (*n* = 3–5) were challenged with a sterile or a MRSA (USA300 LAC::lux)-contaminated transtibial implant, and the infected tibiae were harvested at the indicated times to measure IL-27 protein levels in bone tissue homogenates via ELISA. **b** Cultured RAW264.7 cells and primary murine bone marrow-derived macrophages differentiated with PBS, IFN-γ (50 ng·mL^−1^) or IL-4 (20 ng·mL^−1^) to generate M0, M1 and M2 macrophages, respectively, were exposed to *S. aureus* USA300 for 24 h (MOI = 1, 10, 50). Subsequently, culture supernatants were collected and assessed for IL-27p28 via ELISA. **c** Cultured murine calvarial MC3T3-E1 osteoblasts and primary bone marrow-derived osteoblasts were exposed to *S. aureus* USA300 for 24 h (MOI = 1, 10, 50), and culture supernatants were assessed for IL-27 via ELISA. **d** Cultured murine primary bone marrow-derived osteoclasts were exposed to *S. aureus* USA300 (MOI = 1, 10, 50), and supernatants were assessed for IL-27p28 via ELISA. The data from each experiment are presented with the mean ± SD for the group (*n* = 3–5) (*P < 0.05, **P < 0.01, ***P < 0.001 ****P < 0.000 1, ANOVA)

Given the association between IL-27 expression and *S. aureus* osteomyelitis in patients, we next measured IL-27 in mice with *S. aureus* osteomyelitis induced using a well-established transtibial model^32–36^. C57BL/6 (WT) and IL-27Rα^−/−^ mice were challenged with bioluminescent MRSA (USA300 LAC::lux), and tibiae were harvested 14 days post-infection to assess the bacterial load and measure IL-27 levels via ELISA. We observed early induction of IL-27 expression in tibiae due to *S. aureus* infection, and this response was autoregulated by IL-27/IL-27R signaling, as IL-27 induction on Day 1 was not observed in IL-27Rα^−/−^ mice (Fig. 2a, *P* < 0.05). The ex vivo CFUs in the implants, tibiae, and soft tissues were not different in IL-27Rα^−/−^ mice compared to WT animals (Fig. S1), suggesting that IL-27 is dispensable for clearance of *S. aureus* from bone.

### *S. aureus* induces IL-27 secretion in macrophages and osteoblasts but not in osteoclasts

Although early induction of IL-27 by *S. aureus* infection of bone was evident, its cellular origin was unclear. Thus, to assess the potential of different cell populations to produce IL-27 in response to *S. aureus*, we measured IL-27 production by murine osteoblasts, osteoclasts, and macrophages in vitro. *S. aureus* induced significant concentration-dependent IL-27 secretion 24 h post-infection in both RAW 264.7 macrophages and M0, M1, and M2 murine macrophages (Fig. 2b, *P* < 0.05). Surprisingly, murine calvarial MC3T3-E1 osteoblasts and primary bone marrow-derived osteoblasts produced significantly higher amounts of IL-27 at 24 h post-infection (Fig. 2c, *P* < 0.05). In sharp contrast, bone marrow-derived osteoclasts did not produce IL-27 when exposed to *S. aureus* in vitro (Fig. 2d, *P* < 0.05). To our knowledge, this is the first demonstration of IL-27 expression by osteoblasts and suggests an important early host innate immune response against *S. aureus* infection.

### Systemic IL-27 inhibits draining abscess formation and bone loss during the establishment of *S. aureus* osteomyelitis

**Fig. 3:**
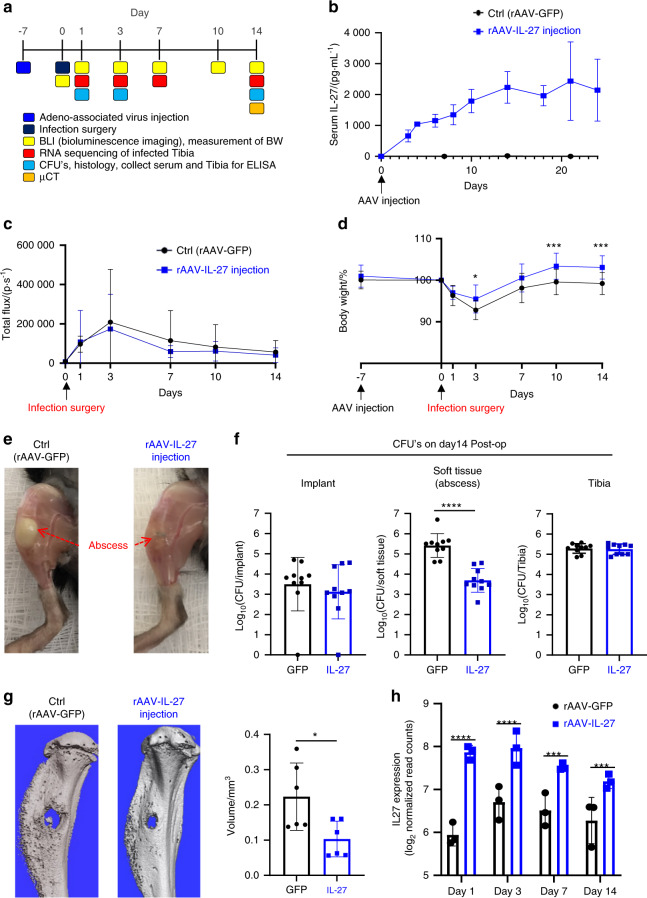
Systemic IL-27p28 induces local IL-27 expression and ameliorates soft tissue infection at the surgical site and osteolysis during *S. aureus* implant-associated osteomyelitis. **a** Schematic illustration of the experimental design in which 8-week-old female C57BL/6 WT and IL-27Rα^−/−^ mice (C57BL/6J background) received an injection of rAAV-IL-27p28 or rAAV-GFP in the quadriceps 1 week prior to challenge with a MRSA (5 × 10^5^ CFU of USA300 LAC::lux)-contaminated transtibial implant. The time points for the various outcome measures are also indicated. **b** WT mice were infected with 0.5 × 10^12^ genome copies/mouse rAAV-IL-27p28 (*n* = 3) or rAAV-GFP (control, *n* = 5) via intramuscular injection, and serum samples were collected longitudinally to assess IL-27 levels via ELISA. The exogenous IL-27p28 levels in serum are presented as the means ± SDs. **c**–**f** A separate cohort of mice were infected with rAAV-IL-27p28 or rAAV-GFP via injection and subjected to transtibial implant challenge (*n* = 16). The longitudinal BLI (**c**) and total body weight (**d**) values are presented as the means ± SDs (*P < 0.05 on Day 3, ***P < 0.001 on Day 10 and 14, two-way ANOVA). **e** Photographs of representative infected tibiae obtained on Day 14 are presented to show the large vs. small draining abscesses observed in rAAV-GFP- vs. rAAV-IL-27p28-treated mice. The mice were euthanized on Day 14, and the infected tibiae were harvested for CFU (**f**) and micro-CT (**g**) analyses. CFUs in the implant, soft tissue and tibia were quantified, and the data for each tibia are presented with the mean ± SD for the group (*n* = 10, ****P < 0.000 1, *t*-test). Re*p*resentative 3D renderings of extensive peri-implant osteolysis and reactive bone formation in rAAV-GFP- vs. rAAV-IL-27p28-treated tibiae are shown with volumetric bone loss in infected tibiae. Data are presented for each tibia with the mean ± SD for the group (*n* = 6, *P < 0.05, *t*-test). **h** Total RNA was extracted from infected tibiae (*n* = 3) at different time points, and *IL-27* mRNA levels were measured by bulk RNA sequencing. The expression data are presented as the normalized read counts with the mean ± SD for the group (****P < 0.000 1 on Days 1 and 3, ***P < 0.001 on Days 7 and 14)

We next examined whether systemic IL-27 mediates bacterial clearance during *S. aureus* osteomyelitis in our murine model. MRSA (USA300 LAC::lux) was used to induce transtibial osteomyelitis in mice after intramuscular (IM) injection of recombinant adeno-associated virus expressing GFP (rAAV-GFP, control) or rAAV-IL-27p28 (Fig. 3a). We first confirmed the magnitude and stability of IL-27 production after rAAV-IL-27p28 injection and detected a temporal increase in serum IL-27, which peaked (~2 ng·mL^−1^) on Day 24 post-injection (Fig. 3b). While rAAV-IL-27p28 treatment did not show an effect on in vivo *S. aureus* growth, as assessed by the bioluminescence intensity (BLI) values (Fig. 3c), rAAV-IL-27p28-treated mice showed improved body weight recovery following septic surgery compared to rAAV-GFP-treated animals (Fig. 3d, *P* < 0.05). Remarkably, mice injected with rAAV-IL-27 showed much smaller draining abscesses at the sites of bone infection (Fig. 3e). Ex vivo CFU analysis confirmed that the bacterial load in surgical site soft tissues was significantly lower in rAAV-IL-27p28-treated mice (Fig. 3f, *P* < 0.05). Moreover, high-resolution μCT demonstrated that peri-implant osteolysis was decreased in mice treated with rAAV-IL-27p28 compared to mice receiving rAAV-GFP (Fig. 3g).

MRSA-infected tibiae from rAAV-IL-27p28- and rAAV-GFP-treated mice were harvested on Days 1, 3, 7, and 14 post-septic surgery and subjected to bulk RNA sequencing. As expected, IL-27 expression in the infected tibiae was significantly upregulated in mice receiving rAAV-IL-27p28 compared to mice receiving rAAV-GFP at all time points (Fig. 3h), suggesting a positive feedback effect^37^. To validate the transcriptome data, the IL-27p28 protein level was measured at early time points in mouse tibia homogenates using ELISA (Fig. S2). Collectively, these results demonstrated that IL-27 affects abscess formation and bone osteolysis. Of note, CFU quantification in the implants revealed similar bacterial loads between the groups, suggesting that systemic IL-27 treatment does not affect biofilm formation on implants. Indeed, scanning electron microscopy (SEM) confirmed these findings (Fig. S3).

### IL-27 enhances the accumulation of RORγt^+^ neutrophils at early stages of implant-associated osteomyelitis

**Fig. 4:**
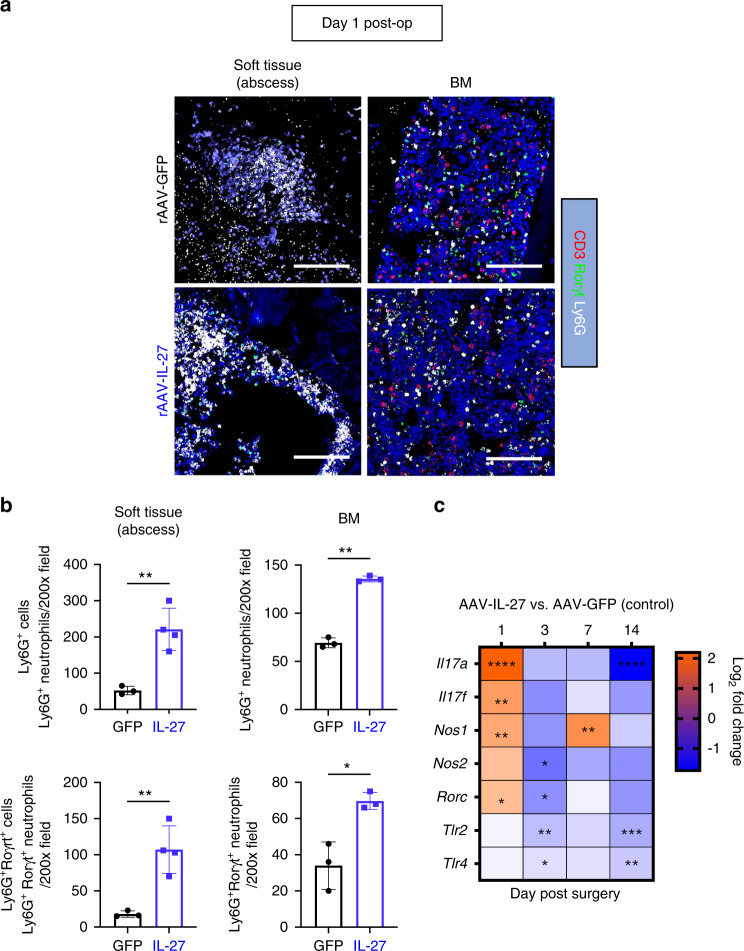
Systemic IL-27p28 enhances the accumulation of RORγt^+^ neutrophils at the site of infection in mice with implant-associated *S. aureus* osteomyelitis. **a** Tibiae from WT mice infected with rAAV-GFP or rAAV-IL-27p28 and challenged with a MRSA-contaminated implant as described in Fig. 3 were harvested on Day 1 post-surgery and processed for multiplex immunofluorescence microscopy. Representative images of tibial sections containing abscesses adjacent to the pin and noninvolved bone marrow (BM) on Day 1 post-surgery stained for Ly6G^+^ (white), RORγt (green), and CD3^+^ (red) are shown at 200× magnification (scale bar = 100 µm). **b** Histomorphometric analysis was performed to quantify the numbers of Ly6G^+^ and Ly6G^+^RORγt^+^ neutrophils per 200× field in soft tissue and BM, and the data are presented with the mean ± SD for the group (*n* = 3 or 4, *P < 0.05, **P < 0.01, *t*-test). **c** Gene expression profiles associated with neutrophil migration are shown on a heatmap with log_2_ fold change values on each day in mice treated with rAAV-IL-27p28 vs. rAAV-GFP. Orange and blue indicate upregulation and downregulation, respectively (*n* = 3, log_2_ fold change, *P < 0.05, **P < 0.01, ***P < 0.001, ****P < 0.000 1)

We next explored possible scenarios that can lead to the observed suppression of *S. aureus* Staphylococcus abscess communities (SACs) and reduced bone loss at the surgical site. Immunohistopathology of infected tibiae revealed an increased number of neutrophils (Ly6G^+^ cells) on Day 1 post-surgery in both SACs and the adjacent bone marrow in rAAV-IL-27p28-treated animals compared to rAAV-GFP control animals (Fig. 4a). Histomorphometric quantification confirmed the significant increase in neutrophils in SACs and in the bone marrow in the rAAV-IL-27 group (Fig. 4b, *P* < 0.05). Interestingly, increased numbers of Ly6G^+^RORγt^+^ neutrophils were observed in the rAAV-IL-27p28 group (Fig. 4a, b, *P* < 0.05), suggesting a possible involvement of proinflammatory IL-17 signaling in bacterial clearance. Indeed, transcriptome analyses revealed increased expression of proinflammatory *IL17A*, *IL17F*, and *RORC* (which encodes the IL-17 transcription factor RORγt^38^) genes in rAAV-IL-27p28-treated mice early during infection (Fig. 4c). As expected, these genes were downregulated at the later stages of infection due to reduced bone disease. Similar trends were observed for immunostimulatory genes associated with Toll-like receptor (TLR) and iNOS signaling, suggesting an IL-27-mediated proinflammatory innate response (Fig. 4c). Indeed, we confirmed that stimulation with a combination of IL-27 and the TLR agonist lipopolysaccharide (LPS) increased nitric oxide (NO^-^) production in primary macrophages, revealing the synergistic properties of IL-27 in enhancing TLR-driven production of microbicidal nitric oxide (Fig. S4).

### Systemic IL-27 reduces bone loss and osteoclast formation during implant-associated osteomyelitis

**Fig. 5:**
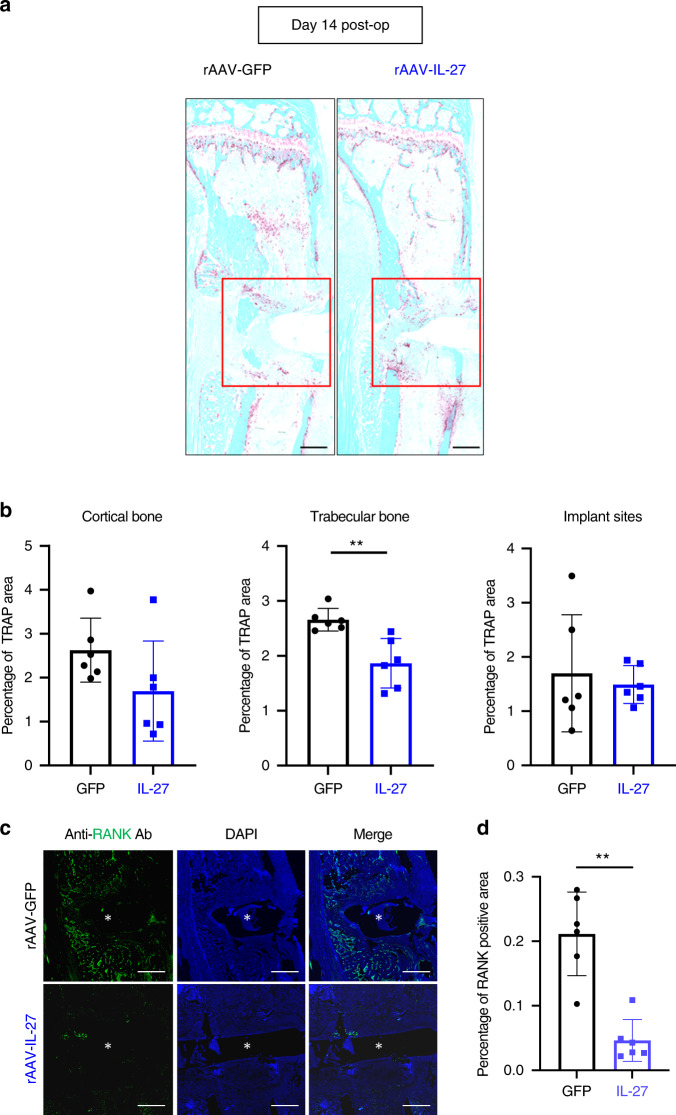
Systemic IL-27p28 impairs osteoclast formation during implant-associated osteomyelitis. **a** Tibiae from the mice described in Fig. 3 were processed for histological staining for tartrate-resistance acid phosphatase (TRAP). Representative images at 2× magnification are shown (scale bars = 500 µm). **b** The TRAP-stained areas within the cortical bone regions, trabecular bone regions, and implant sites (red box) were quantified as percentages, and the data are presented for each tibia with the mean ± SD for the group (*n* = 6, **P < 0.01, *t*-test). **c** Tibia sections were also processed for immunofluorescence histochemistry for RANK, and representative images at 200× magnification are shown (scale bar = 500 µm). The white asterisk indicates the hole where the L-shaped pin was inserted. **d** The RANK-stained area in the bone marrow surrounding the infection site was quantified as a percentage, and the data are presented for each tibia with the mean ± SD for the group (*n* = 6, **P < 0.01, *t*-test)

μCT demonstrated that peri-implant osteolysis was ameliorated in infected mice subjected to rAAV-IL-27p28 treatment (Fig. 3g). We hypothesized that the decreased bone loss was due to reduced osteoclast formation and differentiation. Histomorphometric analysis of tibial sections stained for tartrate-resistant acid phosphatase (TRAP) and immunostained for RANK confirmed decreased osteoclast formation and resorption of trabecular bone due to systemic IL-27 treatment (Fig. 5b, c).

### The effects of IL-27 on *S. aureus* osteomyelitis are dependent on the IL-27/IL-27R axis

**Fig. 6:**
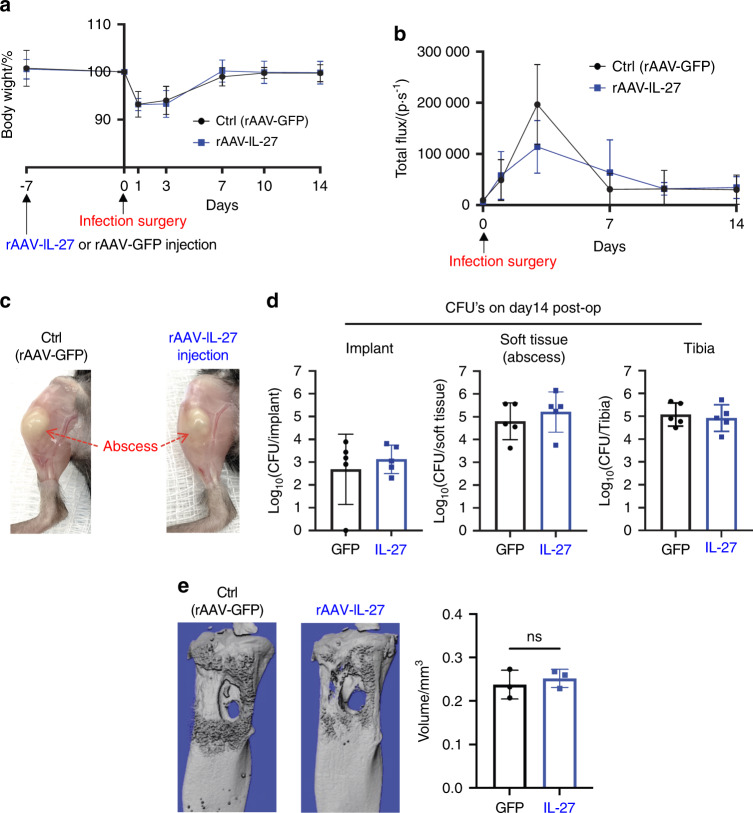
The effects of IL-27 on implant-associated osteomyelitis depend on IL-27Rα expression. Female IL-27Rα^−/−^ mice (*n* = 5) were injected with rAAV-IL-27p28 or rAAV-GFP and challenged with a MRSA-contaminated transtibial implant as described in Fig. 3. Animal weights (**a**) and BLI values (**b**) were measured on Days 0, 1, 3, 7, 10 and 14, and the data are presented as the mean ± SD for the group (*n* = 5). **c** Representative photographs acquired on Day 14 post-surgery show similar large draining abscesses in both groups. **d** CFUs in the implant, surgical site soft tissue, and tibia were quantified after euthanasia on Day 14 post-surgery, and the data for each tibia are presented with the mean ± SD for the group (*n* = 5). **e** Peri-implant osteolysis on Day 14 was evaluated by μCT analyses (*n* = 3). No differences were observed between the experimental groups

It is plausible that IL-27 could directly be involved in the recruitment of neutrophils to sites of *S. aureus* infection. Thus, we examined whether IL-27 is chemotactic for neutrophils. An in vitro chemotaxis assay using granulocytic HL-60 cells revealed that IL-27 did not promote the migration of granulocytes through the Boyden chamber membrane (Fig. S5). IL-27 was also not chemotactic for primary bone marrow-derived macrophages (data not shown). Alternatively, it is possible that IL-27/IL-27R signaling could extrinsically induce chemotaxis of innate immune cells to the infection site. Therefore, we repeated the in vivo *S. aureus* osteomyelitis experiments using IL-27 receptor α knockout (IL-27Rα^−/−^) mice. Fourteen days post-infection, the body weight changes (Fig. 6a) and BLI values (Fig. 6b) were similar between IL-27Rα^−/−^ mice treated with rAAV-IL-27p28 and those treated with rAAV-GFP. Most interestingly, the ex vivo CFUs in the implants, surgical site soft tissues, and tibiae were similar in IL-27Rα^−/−^ mice (Fig. 6d). Furthermore, no differences were detected between the groups in the formation of draining abscesses on these implants (Fig. 6c) or in peri-implant osteolysis (Fig. 6e). These data indicate that the effects of rAAV-IL-27p28 on *S. aureus* osteomyelitis in WT mice are mediated by IL-27/IL-27R signaling.

## Discussion

Cytokines, including IL-27, are central for the timely induction of immune responses during infection. Thus, elucidation of the functions of IL-27 in the context of infection is essential to improve our understanding of protective vs. pathogenic host immunity^21^. In this study, we measured systemic levels of IL-27 in serum from patients with *S. aureus* osteomyelitis. These clinical studies revealed elevated serum IL-27 levels in patients with *S. aureus* bone infections. In mice, we demonstrated that IL-27 expression is induced early during *S. aureus* infection in macrophages and osteoblasts. Remarkably, exogenous IL-27 treatment decreased the severity of *S. aureus* osteomyelitis, including reductions in abscess formation and bone loss. The observed phenotype was likely linked to an IL-17-mediated proinflammatory neutrophil response.

A notable finding of our study is that serum IL-27 levels were highly associated with *S. aureus* osteomyelitis in patients (AUC = 0.922). Previous studies have shown that serum IL-27 levels are elevated in sepsis patients, indicating the potential of this cytokine as a diagnostic biomarker for sepsis^15–19,39^. A single-center prospective study demonstrated that serum IL-27 levels had an AUC of 0.75 in patients with sepsis^17^. Although IL-27 levels were 60-fold higher in patients immediately following septic death than in uninfected patients, we could not calculate AUC values due to the low number of patients with septic death. However, our study indicates that IL-27 could be an diagnostic marker associated with *S. aureus* osteomyelitis and could help to predict septic complications. However, extensive patient cohort studies are required to formally assess its diagnostic potential.

*S. aureus* can persist intracellularly in osteoblasts, contributing to chronic osteomyelitis^40–42^. This persistence can lead to the induction of osteoclastogenic and inflammatory cytokines^43–47^, osteoblast apoptosis^48^, and increased antibiotic tolerance^41,49,50^. A remarkable finding of our study is that *S. aureus* induces significant levels of proinflammatory IL-27 expression in osteoblasts but not in osteoclasts. Osteoclasts, although capable of intracellular *S. aureus* uptake, exhibit diminished bactericidal activity compared to that of bone marrow-derived innate immune cells^51^. Moreover, we observed that exogenous expression of IL-27 contributes to increased neutrophil accumulation and impaired osteoclast formation in the bone marrow milieu. Perhaps IL-27 treatment could promote active *S. aureus* uptake by osteoblasts and bone marrow innate immune cells rather than by osteoclasts. However, our results indicate an important role for this cytokine in orchestrating bone homeostasis and remodeling during osteomyelitis.

*S. aureus*-infected tibiae treated with rAAV-IL-27p28 exhibited substantial accumulation of neutrophils expressing RORγt, which are an innate source of IL-17^52^, and induction of IL-17, TLR, iNOS signaling genes early during infection. These results indicate an IL-27-mediated early innate immune response driving bacterial clearance in these animals. However, transcriptomic analyses also revealed suppression of these genes at later time points during the chronic phase. From these observations, it is conceivable that IL-27 exhibits time-dependent functions in host immunity, ranging from protective immunity in acute *S. aureus* osteomyelitis to suppressive immunity during chronic infection. A recent study using a murine model of intrafemoral osteomyelitis demonstrated similar time-dependent changes in the host response during *S. aureus* osteomyelitis using gene expression analyses^53^.

IL-27 is known to directly inhibit the early stages of RANKL-induced osteoclastogenesis and suppress osteoclast formation^54–59^. Here, we observed that systemic rAAV-IL-27p28 treatment led to impaired osteoclast formation and differentiation, suppressed RANK signaling, and reduced bone osteolysis in *S. aureus*-infected mice. IL-27 expression studies in other disease models have shown similar marked reductions in bone loss^60,61^. In murine models of collagen-induced arthritis, researchers observed that IL-27 was expressed in rheumatoid arthritis synovial membranes and that ectopic IL-27 expression decreased disease severity compared to that in untreated control mice^60,61^. In our study, it is conceivable that the observed reduction in bone loss in rAAV-IL-27p28-treated mice was due to reduced infection and not due to the direct suppressive effects of IL-27 on osteoclast formation. Additional studies at the cellular level are required to confirm the effects of IL-27 on osteoclastogenesis during *S. aureus* osteomyelitis.

Systemic IL-27 delivery led to amelioration of soft tissue infection at the surgical site and peri-implant bone loss in animals with *S. aureus* osteomyelitis. However, the bacterial loads in the implant and bone were not affected by IL-27 delivery, underscoring the ability of *S. aureus* to invade deep within the immune-privileged environment of bone^62^. Interestingly, the effects of IL-27 on abscess formation and bone osteolysis were lost in IL-27 receptor α knockout mice, suggesting a direct role of IL-27/IL-27R signaling in modulating immune and bone cell functions. Similarly, Wang et al. showed that administration of recombinant IL-27 improved bacterial clearance and host survival in a rodent model of *Clostridium difficile* infection^63^. In contrast, other studies reported that IL-27 blockade increased the severity of sepsis-induced myocardial dysfunction in an endotoxic shock syndrome murine model^64^. Collectively, these studies highlight the diverse effects of IL-27 in various bacterial infections.

**Fig. 7:**
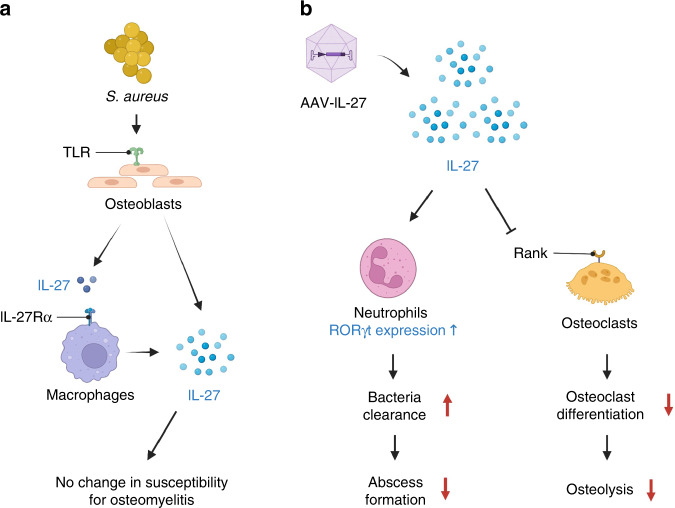
Schematic model of IL-27-mediated immune regulation during *S. aureus* osteomyelitis. **a** *S. aureus* induces IL-27 expression via TLR activation in osteoblasts and macrophages early during the onset of osteomyelitis. Secretion of IL-27 is dependent on the autoregulatory IL-27 signaling pathway. However, endogenous IL-27 does not influence host susceptibility to osteomyelitis. **b** On the other hand, expression of IL-27 due to administration of rAAV-IL-27p28 at supraphysiologic levels induces the accumulation of proinflammatory IL-17-producing RORγt^+^ neutrophils, leading to decreased abscess formation and increased bacterial clearance at the site of infection. Furthermore, the abundance of IL-27 suppresses RANK expression and inhibits osteoclast differentiation, leading to decreased bone osteolysis during chronic osteomyelitis

To summarize our findings, we propose a schematic model of IL-27-mediated immune regulation during *S. aureus* osteomyelitis (Fig. 7). At the onset of *S. aureus* osteomyelitis, osteoblasts and macrophages induce IL-27 secretion via TLR activation, and this process is dependent on the autoregulatory IL-27/IL-27Rα signaling pathway. However, endogenous IL-27 is not sufficient to influence host susceptibility to osteomyelitis. In sharp contrast, exogenous expression of IL-27 induces the accumulation of proinflammatory IL-17-producing RORγt^+^ neutrophils, which leads to decreased abscess formation and increased bacterial clearance at the infection site. Furthermore, the abundance of IL-27 suppresses RANK expression and inhibits osteoclast differentiation, leading to decreased bone osteolysis during chronic osteomyelitis. The proposed model of IL-27-mediated immune homeostasis is preliminary and warrants several future investigations. First, we utilized an AAV-IL-27p28 monomer in our studies, which could exert effects on IL-27R signaling that differ from those of the IL-27 heterodimer (p28+ EBI3)^21,65–68^. Understanding the potential differential effects of the monomer and heterodimer in the context of *S. aureus* osteomyelitis is an important future consideration. Second, we need to examine the complex temporal changes in the infiltrating immune cells in the bone marrow niche, which contribute to IL-27/IL-27R crosstalk during *S. aureus* osteomyelitis. Third, we need to examine specific chemotactic mechanisms that lead to the infiltration of RORγt^+^ neutrophils into the infection site. Finally, we need to understand how IL-27 prevents cytokine storm and internal organ tissue damage during chronic *S. aureus* osteomyelitis in a more relevant murine model of osteomyelitis-induced sepsis. Humanized mice, which are more susceptible to osteomyelitis-induced sepsis caused by MRSA, may be better suited for these studies^69^. These studies will further our understanding of IL-27/IL-27R signaling during *S. aureus* osteomyelitis.

## Materials and methods

### Bacterial strains

Methicillin-resistant *S. aureus* (USA300 LAC) was used for all in vitro experiments, and a bioluminescent strain of USA300 (USA300 LAC::lux) was used for all in vivo experiments, as previously described^32–34,36,69^.

### Ethics statement and patient enrollment

Serum samples were collected from *S. aureus* osteomyelitis patients (*n* = 23) and uninfected patients undergoing elective total joint replacement (*n* = 10). Additionally, serum samples were collected immediately postmortem in patients who succumbed to *S. aureus* osteomyelitis sepsis (*n* = 5). All recruited patients were either enrolled in an international biospecimen registry (AO Trauma Clinical Priority Program (CPP) Bone Infection Registry)^70^ or participated in IRB-approved clinical studies conducted at Virginia Commonwealth University. Patients were recruited with local IRB approval at various institutions, and patient information was collected in a REDCap database managed by AO Trauma and VCU data management administrators. Laboratory investigators had access only to deidentified clinical data, which was provided on request by the data management teams. All ex vivo and in vivo mouse infection studies were performed at the University of Rochester in accordance with protocols approved by the Institutional Animal Care and Use Committee at the university.

### In vitro IL-27 induction assay in osteoblasts, osteoclasts, and macrophages

Primary bone marrow-derived macrophages (BMDMs), osteoblasts, and osteoclasts were generated using precursor cells from bone marrow of the femora and tibiae of 12-week-old female C57BL/6J mice (The Jackson Laboratory). After harvesting, mouse femora and tibiae were washed in RPMI 1640 + 10% FBS, 1% HEPES, and 1% antimicrobial/antimycotic (R10) medium before disinfection with 70% ethanol. Next, the epiphyses of the long bones were cut off, the marrow was flushed out with a 23 G needle, and the bones were resuspended in R10 medium to thoroughly separate the bone and bone marrow. To isolate osteoblasts, bones were cut into small pieces and incubated in αMEM containing 10% FBS, 2 mmol·L^−1^ L-glutamine, 1% antimicrobial/antimycotic, and collagenase I (1 mg·mL^−1^, Thermo Fisher Scientific) for 90 min at 37 °C. After the digestion process, the bone pieces were rinsed to remove unwanted marrow cells, transferred into a flask containing αMEM supplemented with 10% FBS, 2 mmol·L^−1^ L-glutamine, and 1% antimicrobial/antimycotic, and incubated at 37 °C in 5% CO_2_. The migration of osteoblasts from the bone pieces was confirmed after 3 or 4 days. To ensure cellular purity, only primary osteoblasts obtained after three to five passages were used for the IL-27 induction assays. Murine calvarial MC3T3-E1 osteoblasts were plated in DMEM + 10% FBS, 2 mmol·L^−1^ L-glutamine, and 1% antimicrobial/antimycotic to 80% confluence. To differentiate osteoclasts, bone marrow hematopoietic cells were cultured in medium containing macrophage colony-stimulating factor (M-CSF; 30 ng·mL^−1^, PeproTech), and RANKL (100 ng·mL^−1^, PeproTech) was then added and cultured for 5 to 6 days at 37 °C in 5% CO_2_. BMDMs were differentiated with M-CSF (30 ng·mL^−1^, PeproTech). RAW 264.7 cells were cultured in DMEM + 10% FBS, 2 mmol·L^−1^ L-glutamine, and 1% antimicrobial/antimycotic to 80% confluence. Subsequently, BMDMs and RAW 264.7 cells were cultured in R10 medium containing PBS, murine IFN-γ (50 ng·mL^−1^, PeproTech) or murine IL-4 (20 ng·mL^−1^, PeproTech) for 24 h to generate M0, M1, and M2 macrophages, respectively. These cells were then infected with *S. aureus* USA300 at an MOI of 10 for 24 h. Following infection, cell culture supernatants were harvested for measurement of IL-27 secretion using a Mouse IL-27p28 Uncoated ELISA Kit (Invitrogen).

Serum IL-27 concentrations in patients were determined with a Luminex-based Milliplex xMAP Multiplex Assay (Millipore Sigma) according to the manufacturer’s instructions.

### Nitrite production by murine macrophages

Murine BMDMs were pretreated with PBS or murine IL-27 (50 ng·mL^−1^, Biolegend) for 24 h and were then stimulated with or without LPS (100 ng·mL^−1^, Millipore Sigma) to induce reactive nitrogen species production^71^, which is important for the host defense against bacterial infection^72^. Additional experiments were performed utilizing BMDMs stimulated with or without murine IL-27 (50 ng·mL^−1^) for 24 h after pretreatment. Subsequently, nitrite concentrations in the cell culture supernatant were determined with a Griess assay kit (R&D Systems).

### Transwell chemotaxis assay

HL-60 cells (ATCC) were differentiated into granulocytes using 100 mmol·L^−1^ dimethylformamide (DMF) (Millipore Sigma) and plated in the top compartment of Boyden chambers, and a chemotaxis assay was performed according to the manufacturer’s protocol (Millipore Sigma QCM^TM^ Chemotaxis 5 μm 24-Well Cell Migration Assay Kit). Briefly, the chemotaxis of 1 × 10^6^ cells per chamber toward the bottom compartment containing RPMI 1640 medium with or without a chemoattractant [human IL-27 (500 ng·mL^−1^, PeproTech)] or with N-formyl-methionyl-leucyl-phenylalanine (fMLP; 800 ng·mL^−1^, Millipore Sigma) as a positive control was evaluated by incubation for 1 h at 37 °C. After incubation, cell migration into the bottom chamber was quantified as relative fluorescence units (RFUs) according to the manufacturer’s instructions.

### Intramuscular administration of the IL-27-expressing adeno-associated virus vector (rAAV-IL-27)

To achieve sustained exogenous IL-27 expression, mice were injected intramuscularly with recombinant murine IL-27-expressing AAV (IL-27 p28, 0.5 × 10^12^ genome copies per mouse, Vector Biolabs) seven days prior to surgical *S. aureus* infection^65^. AAV was administered adjacent to the left quadriceps muscle, contralateral to the lower limb with the surgical site. Mice injected intramuscularly with AAV expressing recombinant GFP (0.5 × 10^12^ genome copies per mouse, Vector Biolabs) were used as controls.

### Implant-associated MRSA osteomyelitis in mice

The C57BL/6 mice and IL-27Rα-deficient (IL-27R*α*^−/−^) mice on the C57BL/6 background used in the study were purchased from The Jackson Laboratory and maintained in the University of Rochester animal facilities. Our well-validated transtibial implant-associated osteomyelitis model was utilized for all in vivo *S. aureus* challenge experiments in mice^32–34,36,69^. Briefly, L-shaped stainless-steel implants were contaminated with USA300 LAC::lux (5.0 × 10^5^ CFU per mL) grown overnight and surgically implanted into the tibiae of 8-week-old female C57BL/6 mice from the medial to the lateral side. The body weight change and bioluminescence intensity at the infection site were evaluated longitudinally, and terminal assessment of CFUs (in the implant, surgical site soft tissue and tibia), peri-implant osteolysis (high-resolution μCT imaging), biofilm formation on the implant (Zeiss Auriga SEM imaging), and histopathology were performed on Day 14 post-septic surgery, as described previously^32–34,36,69^. Murine infection studies were performed three independent times, and the data from these experiments were pooled.

### Histology

Histopathological analyses were performed according to protocols described previously^32–34,36,69^. Briefly, after μCT, each mouse tibia sample was fixed with 10% formalin neutral buffer for 3 days at room temperature (RT) and then decalcified with 14% ethylenediaminetetraacetic acid disodium salt dihydrate (pH 7.4) for 2 weeks at RT. All samples were embedded in paraffin and sectioned at a thickness of 5 μm. Digital images of the serially stained slides were acquired using a VS120 Virtual Slide Microscope (Olympus, Waltham, MA, USA). To compare the numbers of osteoclasts within infected tibiae, tartrate-resistant acid phosphatase (TRAP) staining was performed. The region of interest (ROI) was manually set around the infection site, as shown in Fig. 5a. The intensity of TRAP staining within each ROI in the infection site and the cortical and trabecular bone regions in each experimental group was quantified using colorimetric histomorphometry with a custom Analysis Protocol Package (APP) in Visiopharm (v.2019.07; Hoersholm, Denmark).

### Multiplex immunofluorescence staining

#### Primary antibodies

The following antibodies were utilized for immunostaining: rabbit anti-TNFRSF11A/RANK (polyclonal, LS-B2077, RRID: AB_1276561, LifeSpan Biosciences) at a 1:20 dilution, goat anti-CD3-epsilon (clone M-20, sc-1127, RRID: AB_631128, Santa Cruz Biotechnology) at a 1:100 dilution, Armenian hamster anti-RORγ (clone RORg2, 646502, RRID: AB_2238503, Biolegend) at a 1:50 dilution, and biotin rat anti-Ly6G (clone 1A8, 127604, RRID: AB_1186108, Biolegend) at a 1:50 dilution.

#### Secondary antibodies

The following antibodies were utilized for immunostaining: Alexa Flour 488-conjugated donkey anti-rabbit IgG (711-546-152, RRID: AB_2340619, Jackson ImmunoResearch Laboratories) at a 1:400 dilution for RANK detection, Alexa Fluor 568-conjugated donkey anti-goat IgG (A-11057, RRID: AB_2534104, Thermo Fisher Scientific) at a 1:200 dilution, FITC-conjugated anti-Syrian hamster IgG (307-096-003, RRID: AB_2339583, Jackson ImmunoResearch Laboratories) at a 1:200 dilution, and Alexa Fluor 680-conjugated streptavidin (S32358, Thermo Fisher Scientific) at a 1:200 dilution.

Then, 5 μm formalin-fixed paraffin sections were incubated at 60 °C overnight for deparaffinization. The tissue sections were quickly transferred to xylene and gradually hydrated by sequential transfer to absolute alcohol, 96% alcohol, 70% alcohol, and finally water. Subsequently, the sections were immersed in Antigen Unmasking Solution (Vector Laboratories) and boiled for 2 h. Nonspecific binding was blocked with 5% normal donkey serum in TBS containing 0.5% Triton X-100 for 40 min at RT in a humidified chamber. Then, primary antibodies at appropriate concentrations were added to these sections and incubated at 4 °C overnight. This step was followed by washing with PBS and incubation with a secondary antibody at RT for 2 h. Finally, the slides were rinsed for 1 h in PBS and mounted with Vectashield antifade mounting medium with DAPI (H-1200, Vector Laboratories, Burlingame, CA, USA). Images were acquired with a Zeiss Axioplan 2 microscope connected to a Hamamatsu camera.

### RNA sequencing of MRSA-infected tibiae

C57BL/6 mice were injected intramuscularly with rAAV-IL-27p28 or rAAV-GFP and then challenged with an *S. aureus*-contaminated transtibial implant as described above. Infected tibiae were collected on Days 1, 3, 7, and 14 post-surgery for RNA sequencing. Tibiae were pulverized in liquid nitrogen (−196 °C) and homogenized using a Bullet Blender Gold instrument (Next Advance). Isolation of total RNA from homogenized tibiae was performed with the TRIzol extraction method (Thermo Fisher Scientific) and RNeasy Mini Kits (Qiagen). Contaminating genomic DNA was removed using TURBO DNase (Thermo Fisher Scientific). A TruSeq Stranded Total RNA Library Prep Gold Kit (Illumina) was utilized for next-generation sequencing library preparation per the manufacturer’s instructions. Libraries were sequenced on the NovaSeq6000 platform (Illumina). Quality filtering and adapter removal were performed with fastp version 0.20.0^73^ using the following parameters: “--in1./$(SAMPLE)_R1.fastq.gz --out1 clt_$(SAMPLE)_R1.fastq.gz --length_required 35 --cut_front_window_size 1 --cut_front_mean_quality 13 --cut_front --cut_tail_window_size 1 --cut_tail_mean_quality 13 --cut_tail -w 8 -y -r -j $(SAMPLE)_fastp.json”. The remaining high-quality processed reads were then mapped to the *Mus musculus* reference genome (GRCm38.p6) with STAR version 2.7.0 f^74^ using the following parameters: “--twopassMode Basic --runMode alignReads --genomeDir $(GENOME) --readFilesIn $(SAMPLE) --outSAMtype BAM Unsorted --outSAMstrandField intronMotif --outFilterIntronMotifs RemoveNoncanonical”. The mapped reads in the GRCm38.p6 gene annotations were counted using the featureCounts read quantification program in Subread version 1.6.4^75^. Then, differential expression analysis and data normalization were performed on each set of raw expression data using DESeq2 version 1.22.1^76^ within R version 3.5.1 with a *P* value threshold of 0.05. All generated sequence data were submitted to Gene Expression Omnibus under accession number GSE168896.

### Statistics

For statistical analyses involving more than two groups, we utilized the nonparametric Kruskal‒Wallis test, one-way ANOVA and two-way repeated measures ANOVA. Unpaired Student’s *t*-test was used to assess the significance of differences between two experimental groups. The data are presented as the means ± standard deviations. A *P*-value of <0.05 was considered significant.

## Supplementary information

Supplemental Figure 1

Supplemental Figure 2

Supplemental Figure 3

Supplemental Figure 4

Supplemental Figure 5

Supplemental figure legend

## Data Availability

All RNA sequence data have been submitted to the Gene Expression Omnibus under accession number GSE168896.
