# Supplementary figures and images for: Systemic IL-27 administration prevents abscess formation and osteolysis via local neutrophil recruitment and activation
Source: Bone Res. 2022 Aug 26;10:56. doi: 10.1038/s41413-022-00228-7 (PMC9418173; doi:10.1038/s41413-022-00228-7)

**A****Implant**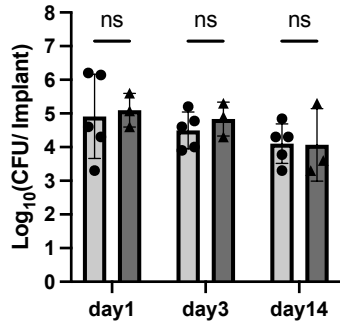**Tibia**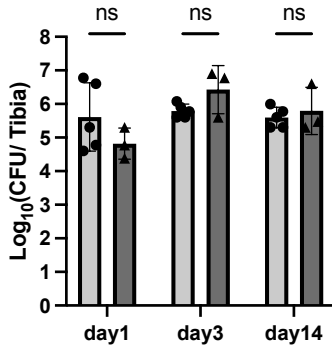

● WT  
▲ IL27Rα<sup>-/-</sup>

**B****Soft tissue  
(Abscess)**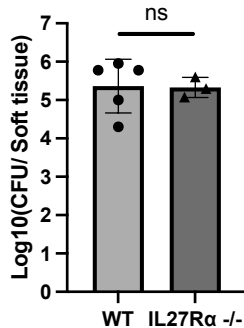

Supplement: Supplementary file 1 — Supplemental Figure 1 [file 41413_2022_228_MOESM1_ESM.pdf]

## Tibia

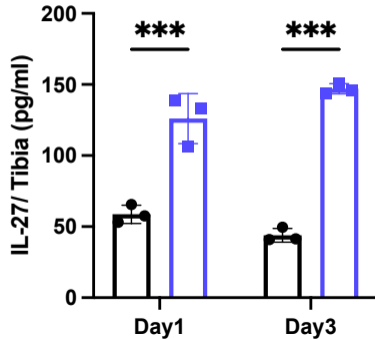

## Serum

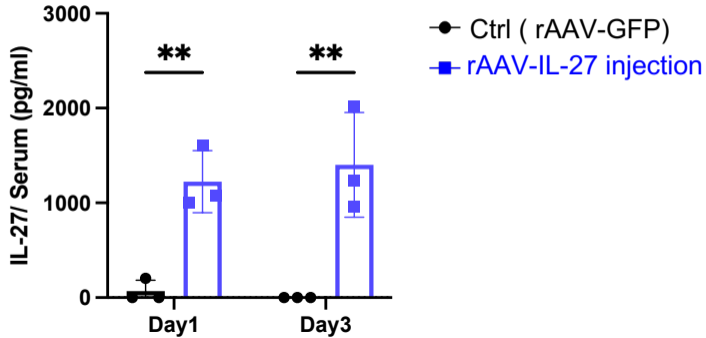

Supplement: Supplementary file 2 — Supplemental Figure 2 [file 41413_2022_228_MOESM2_ESM.pdf]

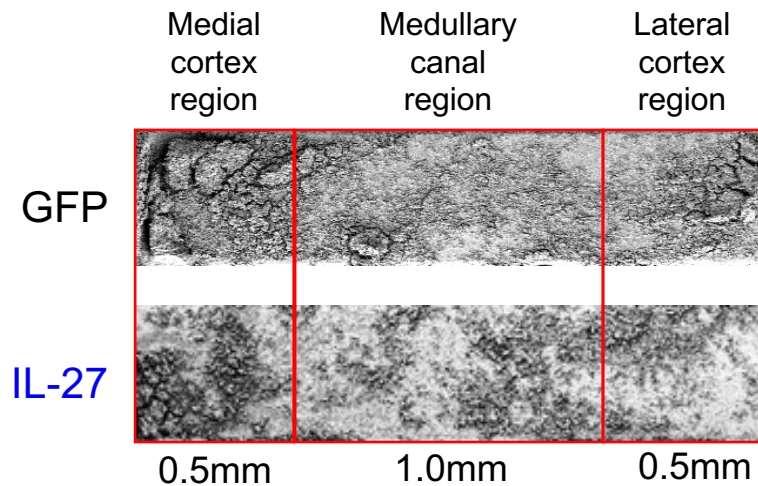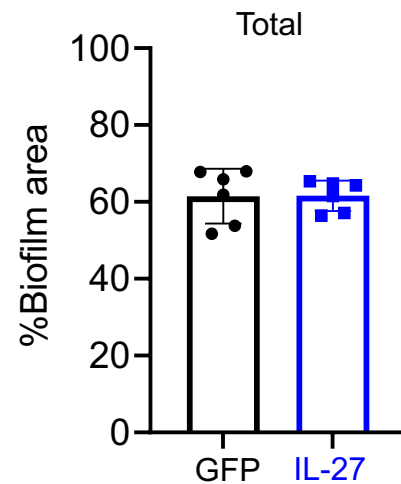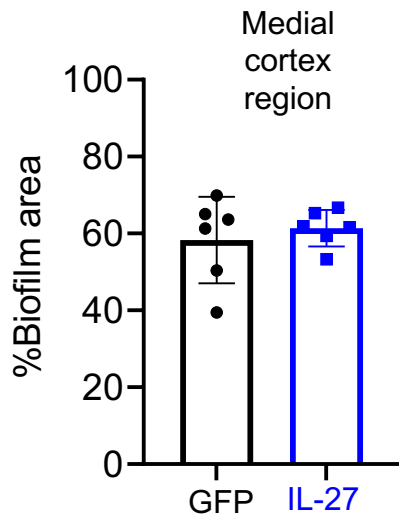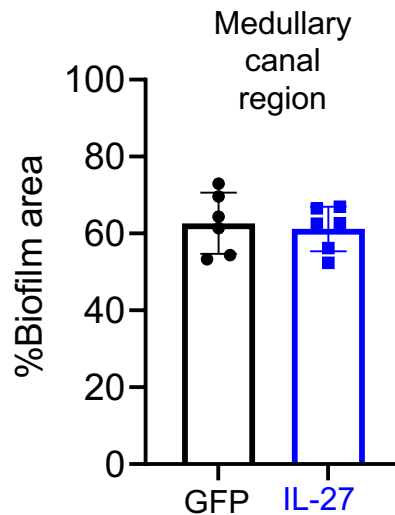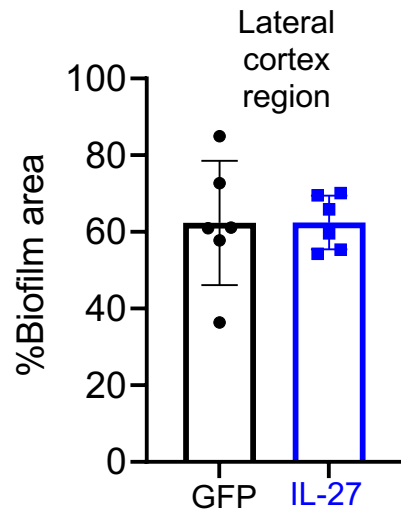

Supplement: Supplementary file 3 — Supplemental Figure 3 [file 41413_2022_228_MOESM3_ESM.pdf]

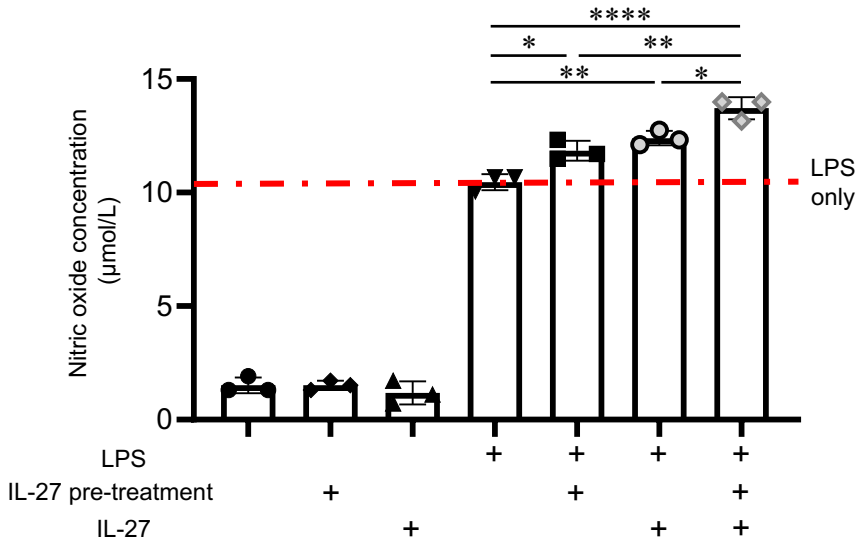

Supplement: Supplementary file 4 — Supplemental Figure 4 [file 41413_2022_228_MOESM4_ESM.pdf]

## IL-27 chemotaxis assay

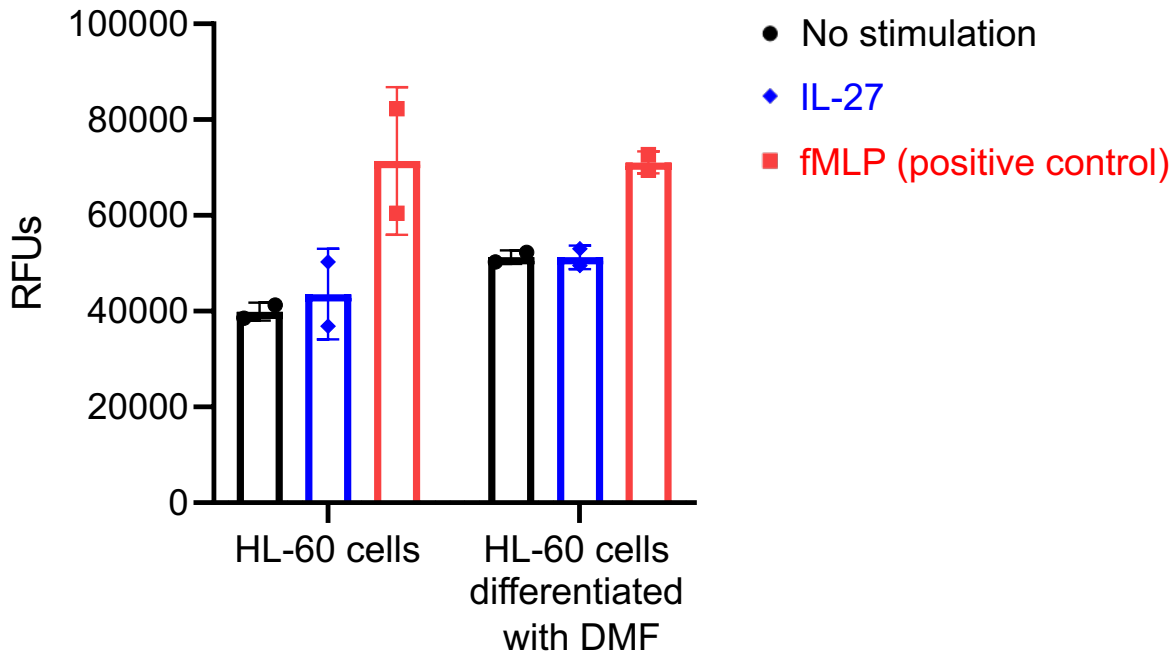

Supplement: Supplementary file 5 — Supplemental Figure 5 [file 41413_2022_228_MOESM5_ESM.pdf]
