# Supplementary material for: Systemic IL-27 administration prevents abscess formation and osteolysis via local neutrophil recruitment and activation
Source: Bone Res. 2022 Aug 26;10:56. doi: 10.1038/s41413-022-00228-7 (PMC9418173; doi:10.1038/s41413-022-00228-7)
Supplement: Supplementary file 6 — Supplemental figure legend [file 41413_2022_228_MOESM6_ESM.docx]

**Supplemental Figure 1. Disruption of the IL27/IL-27Rα axis did not affect susceptibility to *S. aureus* osteomyelitis.** Female WT and IL-27Rα^-/-^ mice (C57BL/6 background) were challenged with a MRSA (USA300 LAC::lux)-contaminated transtibial implant. **(A)** Ex vivo CFU analysis was performed on the implants and tibiae on Days 1, 3, and 14 post-surgery. (n=5 for WT and n=3 for IL-27Rα -/- mice, ANOVA). **(B)** CFUs in surgical site soft tissue were quantified on Day 14 post-surgery (n=5 for WT and n=3 for IL-27Rα -/- mice, t test). The data for each tibia are presented with the mean +/- SD for the group.

**Supplemental Figure 2.** **Systemic AAV-IL-27p28 treatment in mice leads to differential IL-27p28 expression levels in bone and blood.** Female C57BL/6J mice were challenged with a MRSA (USA300 LAC::lux)-contaminated transtibial implant, and tibiae were harvested and homogenized at the indicated time points to assess IL-27 levels using ELISA. The data from each experiment are presented with the mean +/- SD for the group (n=3, ***p*<0.01, ****p*<0.001).

**Supplemental Figure 3. Systemic IL-27p28 does not affect biofilm formation on the implant during *S. aureus* implant-associated osteomyelitis in vivo.** Mice were injected intramuscularly with rAAV-IL-27 or rAAV-GFP and then challenged with a MRSA (USA300 LAC::lux)-contaminated transtibial implant as described in Figure 3. Biofilm formation on the implant was evaluated via SEM and imaging after euthanasia on Day 14 post-surgery. No difference was detected in the area percentages of biofilm formation on the implants between rAAV-IL-27- and rAAV-GFP-challenged mice (n=6).

**Supplemental Figure 4.** **IL-27 enhances LPS-induced nitrite production by macrophages.** Primary bone marrow-derived murine macrophages were pretreated with PBS or IL-27 (50 ng/ml) for 24 hours and then stimulated with LPS (100 ng/ml) in the presence or absence of IL-27 (50 ng/ml) for 24 hours. Nitrite levels in the culture supernatant were determined via a Griess assay, and the data from each experiment are presented with the mean +/- SD for the group (n=3; **p*<0.05, ***p*<0.01, *****p*<0.0001 by one-way ANOVA).

**Supplemental Figure 5. IL-27 does not stimulate myeloid cell chemotaxis.** HL-60 cells were differentiated for 7 days in the presence or absence of dimethylformamide (DMF) (9 µg/ml) and then plated in Boyden chambers. Cell culture medium with or without IL-27 or fMLP (positive control) was placed in the compartments below the chambers and incubated for 1 hour. Subsequently, the cells that migrated in each well were stained with fluorescent dye, and the signal intensity was evaluated using a fluorescence plate reader (n=2). No difference in the chemotactic activity of granulocytes was observed between the experimental groups.
